# Asthma out of control? A structured review of recent patient surveys

**DOI:** 10.1186/1471-2466-6-S1-S2

**Published:** 2006-11-30

**Authors:** Stephen T Holgate, David Price, Erkka Valovirta

**Affiliations:** 1 Infection, Inflammation and Repair AIR Division, Level F, South Block, MP810, Southampton General Hospital, Tremona Road, Southampton, SO16 6YD, UK; 2 Department of General Practice and Primary Care, University of Aberdeen, Foresterhill Health Centre, Westburn Road, Aberdeen AB25 2AY, UK; 3 Turku Allergy Center, FIN-20610 Turku, Finland

## Abstract

**Background:**

An understanding of the needs and behaviors of asthma patients is important in developing an asthma-related healthcare policy. The primary goal of the present review was to assess patient perspectives on key issues in asthma and its management, as captured in patient surveys.

**Methods:**

Local, national, and multinational asthma surveys were reviewed to assess patient perspectives, and where possible healthcare provider (HCP) perspectives, on key issues, including diagnosis, treatment, control, quality of life, and other patient-centered outcomes. Twenty-four surveys, conducted or published between 1997 and 2003 in Europe and North America, were included in this review. Substantial differences among studies prevented a formal meta-analysis; instead, data were pooled to allow for general comparisons and qualitative analysis.

**Results:**

The results indicate that patients' knowledge of the underlying causes of asthma and treatment options remains inadequate. Moreover, patients often tolerate poor symptom control, possess meager knowledge of correct drug usage, and display insufficient adherence to therapy. Many patients have a low expectation of receiving an appropriate therapy or of having a positive encounter with the HCP. Among HCPs, there is evidence of inadequate understanding of disease etiology and poor or unstructured communication with patients, resulting often in inaccurate assessment of disease severity. Moreover, patients often underreport their symptoms and severity, which in turn could lead to misclassification and undertreatment.

**Conclusion:**

Improving patient education about the importance of achieving optimal asthma control, along with improved communication between patients and HCPs, emphasizing treatment options and optimal treatment of inflammation, may lead to better outcomes and improved asthma management in daily practice.

## Background

Asthma is a chronic, inflammatory condition of the airways characterized by airway hyperresponsiveness and episodic respiratory symptoms, such as breathlessness, wheezing, chest tightness, and coughing. The prevalence of asthma continues to increase in many countries: the current estimate of 300 million people with asthma worldwide is expected to increase by 33% to 400 million by 2025 [1,2]. In addition to the economic burden of asthma, which is considerable, there are physical, emotional, and social effects, leading to reduced quality of life (QoL) of patients and their families [3-6].

International surveys have been valuable for understanding and managing asthma. The International Study of Asthma and Allergies in Childhood, the European Community Respiratory Health Survey, and other surveys have provided much needed information on the global patterns of asthma prevalence from childhood to adulthood, and have generated new hypotheses for further testing and validation [2,7-10]. An understanding of the needs and behaviors of asthma patients is also important in developing asthma-related healthcare policy. The primary goal of the present review was to assess patient perspectives on key issues in asthma and its management, including diagnosis, treatment, control, and QoL, as captured in patient surveys in Europe and North America. Where available in the same surveys, healthcare provider (HCP) perspectives on a number of key issues in asthma and its management were assessed. We have attempted to draw conclusions that are relevant and useful to asthma caregivers.

## Methods

A review of asthma patient surveys in Europe and North America, conducted or published between 1997 and 2003, was undertaken by a general multibased literature search using keywords that included 'asthma' and 'survey'; additional searches were performed on key authors' names. The following initial selection criteria were used: observational/experimental survey design, patient-reported outcomes, perceptions or data related to asthma management, and physician-reported aspects of asthma management. From a total of 68 surveys initially screened, 24 surveys (including some substudies of special populations) and analyses were ultimately selected to provide a balanced data pool across a broad range of issues [6,7,11-32]. These included published and unpublished surveys translated from local language when necessary. Unpublished surveys were also provided by the sponsor of this project when there was evidence that they might be relevant, and surveys that were not formally published were supplemented with data on file provided by the sponsor. Some surveys were later excluded if data were not ultimately relevant to the core emergent patterns and questions of the review.

Data from all studies were extracted in a systematic fashion into a large two-dimensional matrix. This approach simplified identification of subsets of surveys in which the methods, including design, populations, and outcomes, were sufficiently similar for possible pooled analysis. The following main criteria were used to structure the matrix: the period of data collection, the nature of data extraction (interview/self-reported questionnaire), questionnaire type (standardized/nonstandardized), location, study objectives, design (cross-sectional/longitudinal), the length of follow-up (if applicable), the sampling method, sample composition and size, the assessment of asthma severity, the types of medication used, the assessment of relevant outcomes, and population demographics.

After the 'extraction phase,' appropriate data were selected and grouped together to strengthen the outcomes measured ('pooling phase'). Key criteria such as the design and sample base were used to group together relevant surveys so as to address the most important outcomes and the incidence of those outcomes (expressed as ranges). At this stage it was clear that substantial differences in studies – which included differences in national or international scope, design, setting, timing, sample bases, and objectives – precluded a formal meta-analysis. Thus, while it was statistically inappropriate to combine results from these surveys, general comparisons were carefully undertaken where there were no confounding factors. A list and overview of the design of surveys reviewed is provided in Table 1.

The review was further classified according to a structured set of most relevant outcomes identified ('assessment phase'). The following patient outcomes were assessed: understanding of the disease (etiology), perception of asthma control, satisfaction with treatment, compliance with therapy, and impact on patient and family QoL. Childhood asthma was examined separately to identify any trends specific to this population, although pediatric data were also included in the wider patient perspective assessment. At the HCP level, the same outcomes that were assessed in patients were evaluated when available; this allowed for a comparison of similarities and differences in perception between HCPs and patients on these issues.

## Results

The 24 surveys reviewed included a total of 66,450 subjects from a total of 24 countries; of this number, 57,817 were patients (including 11,875 children, generally classified as <16 years, and parents of children) and 8,633 were HCPs [6,7,11-32]. Table 1 presents the names of these surveys, together with patient numbers and other descriptors. Table 2 provides a summary of the sample populations contained in these surveys. Table 3 summarizes the general findings from the survey review.

### Asthma understanding and control – the patient perspective

#### Understanding the causes and nature of asthma

A few surveys contained feedback from patients that addressed their disease understanding. In TARGET [11], 38% of patients or parents considered allergy, and 26% airway inflammation, as important to asthma, while 60% were able to specifically name an anti-inflammatory drug and 22% understood that such therapy was needed to reduce or control inflammation associated with their asthma. In a Spanish survey [14], 41% of parents or caregivers were able to distinguish asthma symptoms from those of other respiratory diseases such as bronchitis.

#### Asthma symptoms, and their control and prevention

**Figure 1:**
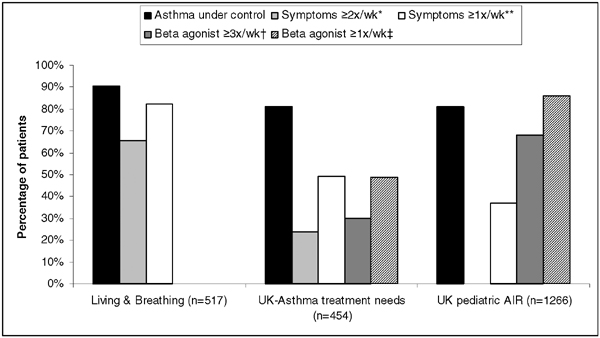
**Patient perceptions of asthma control and symptom frequency**. Percentages of all patients in three UK studies [17,24,25] who considered their asthma to be controlled or well controlled and percentages of patients in those three studies who were experiencing asthma symptoms or using a rescue *β*_2_-agonist. *In the UK Asthma treatment needs study, symptoms were reported ≥3 times per week. **For the UK pediatric Asthma In Real Life (AIR) study, the symptom reported is difficulty breathing. †For the UK pediatric AIR study, the *β*_2_-agonist use reported is two or more times per day. ‡For the UK pediatric AIR study, the *β*_2_-agonist use reported is one or more times per *day*.

Eight surveys [6,11,17,22,24,25,27,32] that collectively included over 15,000 patients provided the core data demonstrating the high levels of symptoms reported by patients, even though they were all receiving asthma therapy. Diurnal symptoms were reported by 46–75% and nocturnal symptoms by 30–70% of the patients in three studies [6,11,22]. Patients often appeared to tolerate high levels of symptoms, often understating the severity of their disease (Figure 1). In three European studies [11,24,25], while a large proportion of patients reported daily symptoms, a greater proportion considered their symptoms under control, and very few considered their disease 'out of control.' One survey reported that a majority of pediatric patients or their parents (65%) considered their asthma well controlled despite a high incidence of breathing difficulties (37%), sleep disturbances (34%), dry cough (29%), and difficulty talking (29%) at least once weekly because of their asthma [17].

Perhaps not surprisingly, some surveys provided data to indicate a poor level of control while others indicated a high level of suboptimal therapy. One survey, comparing treatments across Europe, discovered a complete absence of treatment in <20% to 70% of patients from country to country (70% in Estonia) [23]. In a US survey, only 20% of 2,509 symptomatic patients were found to be using anti-inflammatory therapy (mainly inhaled corticosteroids (ICS)), even though at least 23% of these patients had undergone hospitalization or urgent care during the previous year [27]. In surveys where anti-inflammatory treatment was specified, ICS were commonly used, although in a highly variable range (15–92%). Only 4% of 347 parents of children with asthma in one survey [16] had heard of alternative anti-inflammatory treatments. There was also some evidence for incomplete patient understanding of treatments, with one pan-European survey reporting that, when asked about the definition of controller therapy, an average of 26% of parents of children with asthma gave markedly incorrect responses [12].

#### Expectations and satisfaction

Eighteen surveys, with a combined patient population of approximately 34,000, reported data on patient satisfaction and were pooled for descriptive analysis [6,11,13-17,19-28,32]. Many studies touched on the issue of expectations and satisfaction, sometimes in an oblique way [6,13,15-17,19-22,27]. The general inference from these studies was that large numbers of patients had a low level of satisfaction regarding their contacts with HCPs, and that there was often a low level of patient expectation from therapy. At least two studies showed that optimal therapy is more often provided when patient-physician contact is more frequent and more structured [15,23].

#### Adherence to therapy

Fourteen surveys [6,11-21,26,27] that included approximately 28,300 patients illustrated various adherence issues. A number of patients admitted underuse of their medication (at least 40%) either in terms of frequency or dosing [12,13,15,17-21,26,27]. In several surveys, 40–70% of patients admitted that one factor in their lack of adherence was high discomfort with long-term use of ICS [11,13,14,32]. This was reflected in the observation that approximately one-third of patients reported dissatisfaction with long-term ICS treatment [26]. When patients were prescribed higher doses of ICS by their physicians to regain control, at least 50% refused to adhere to their prescribed therapy fully because of concerns over side effects [13].

#### Impact on lifestyle

Eleven surveys that included approximately 34,700 patients assessed varied lifestyle-related endpoints [6,12-15,17-19,25,27,32]. Overall, these varied data suggest that asthma has a much understated impact on lifestyle and QoL.

Although one survey showed that lifestyle was affected irrespective of disease severity [26], overall the surveys suggested a relationship between severity of disease and QoL. There was some anecdotal indication that, subject to the severity of disease, incomplete control of inflammation and therefore lack of control of asthma symptoms were associated with significantly reduced QoL in patients [6,15,27].

#### Asthma and children

Fifteen surveys [6,11-18,22,25,27,30-32] allowed evaluation of asthma in 8,384 children, some by survey of caregivers. Findings were consistent with those of adult populations, raising particular concerns about high levels of symptoms, dissatisfaction with treatment, impact on lifestyle, concerns related to side effects of treatment, poor compliance, and missed school or work (Table 4).

### Survey contributions by the healthcare provider

Over 8,600 HCPs were included in the surveys reviewed [6,11,16,27,29,30]. Of particular interest was the observation that detailed understanding of asthma pathophysiology, and therefore appropriate treatment, was perhaps limited. In the TARGET survey [11], 59% of 305 physicians believed that allergic factors were dominant in the etiology of asthma, and only 35% (and only 16% of pediatricians) cited underlying airway inflammation. In another survey [27], physicians reported that an average of only 20% of their asthma patients were actually prescribed an anti-inflammatory product (of which 73% received ICS) – usually only after persistent asthmasymptoms during the previous month, and in particular only after an acute event.

Of broad interest was the attitude towards using continued or increased doses of ICS compared with newer therapies. Again, in the TARGET survey [11], although 98% of physicians agreed that it was important to treat inflammation in asthma, ICS were rarely prescribed for periods of greater than 6 months.

### Comparisons of views between patients and healthcare providers

Patients reporting symptoms of asthma often reported that their asthma was well controlled, while in combined patient/HCP surveys their HCP reported that it was not well controlled [13,17,20,21]. Conflicting data, however, were also seen: 1% of patients, as opposed to 7% of their physicians, considered their asthma to be very severe; this contrasted with 1% of patients considering themselves completely free from symptoms, while 24% of the HCPs who assessed them thought the same [21]. In another survey [20], patients were more likely than their physicians to regard their disease as more serious and life-threatening. In the UK Asthma In Real Life study [17], HCPs were also found to underestimate the impact of asthma symptoms on their patients, perhaps as a consequence of patients' willingness to tolerate fairly severe symptoms and associated lifestyle restrictions (with 65% of patients considering themselves to have well-controlled disease despite the high incidence of severe symptoms) and thus not raising them with their HCPs. This survey also found that HCPs underestimated patients' need for relief inhalers. Patients appear to also underreport their incidence of side effects from drugs; for example, direct consultation with 240 patients with asthma also showed they experienced a higher incidence of side effects than their physicians realized [21].

There was a greater agreement in the views of patients and HCPs relating to long-term therapy. In one survey [11], 57% of physicians reported that their patients were reluctant to take corticosteroids long term and their patients agreed: almost 50% and 70% were apprehensive about taking ICS and oral corticosteroids, respectively, generally because of concern over long-term side effects associated with these drugs. In another survey [13], 59% of physicians and 53% of patients were worried about long-term use of corticosteroids, regardless of disease severity.

## Discussion

The present review of 24 patient surveys indicates that patients often experience high levels of asthma symptoms even while receiving asthma therapy. Patients' perceptions of asthma control and prevalence of symptoms were often mismatched: patients who considered their asthma well controlled still reported daily symptoms. Furthermore, while many patients were conscious of the clinical factors indicative of lack of symptom control (coughing, breathlessness, and sleep disturbances), many failed to fully understand the impact of the disease on their everyday life, and patient awareness did not necessarily translate into an understanding of the need to control symptoms. A substantial majority of patients appeared to tolerate suboptimal treatment. In addition, the survey results suggest that asthma may have a substantially underestimated impact on QoL, expressed in terms of difficulty in breathing, ability to exercise, and quality of sleep.

Adherence failure was common because of a variety of factors and not just because of concerns about drug side effects. This was often compounded by a low expectation of receiving an appropriate therapy or of having a positive encounter with the HCP. Surprisingly, physicians were also often unaware of the extent of their patients' poor adherence or misuse of treatments. Moreover, patients often underreported their symptoms and severity, which in turn could have led to misclassification and undertreatment.

Overall, the results of the present review indicate that many patients lack an understanding of the importance of inflammation in asthma, and few are aware of the anti-inflammatory effects of treatments. A recent review of the Asthma Insights and Reality surveys referred to the low level of use of anti-inflammatory medication, the suboptimal control of asthma, and the consequent deterioration in the lifestyle of patients [33]. These results reinforce the need for renewed efforts to effectively inform patients, both through and apart from their HCP. Such general patient-awareness education should emphasize not only the inflammatory etiology of asthma and the importance of appropriate treatment, but also aspects of improved patient self-diagnosis, awareness of additional treatment choices that could further modify disease progression, and also the implications of treatment, such as drug side effects.

Overall, pediatric patients seemed to be better managed than adults with asthma, not simply because there tends to be a stronger emphasis on childhood asthma in most countries, but also because caregivers and parents seemed to show greater involvement in managing their children's disease and were highly conscious of drug safety issues. The surveys did, however, highlight parents' confusion about, and their lack of understanding of, childhood asthma and its therapies. A number of parents (approximately 40%) in one survey regarded asthma in their children as a stigma and were in potential denial of their children's condition [14]. This clearly represents an opportunity for further patient and caregiver education.

As with the adult populations, there was a strong difference between perception of control (by children and their caregivers) and the practical issues of symptom management. In general, children utilized a proportionately greater share of resources than adults [32]. A recent review of epidemiological surveys and clinical studies of asthma in children [34] reports evidence of poor asthma control in children: only a small proportion of children worldwide attain the goals of good asthma control as set out by the Global Initiative for Asthma [35,36].

A common theme that emerges from many of the surveys is the dissatisfaction and poor outcomes seen with asthma controller medications. Among the numerous reasons for this are concerns over safety, poor efficacy, and poor adherence. Encouragingly, both patients and their HCPs generally seemed to agree within the same survey that there was a need to use medications with fewer side effects and fewer long-term complications. There was, however, some evidence of differences in opinion among physicians on the best strategy for long-term and more complete asthma control.

Our report evaluates asthma management from the patients' perspective. The 24 surveys reviewed illustrate the burden of asthma in patients and attitudes toward its diagnosis and treatment among patients as well as HCPs, including asthma specialists, pediatricians, general practitioners, pharmacists, and nurses. A limitation of the present review is therefore that it constituted a qualitative analysis and hence could be considered a subjective appraisal. A true quantitative meta-analysis was not possible because of the variety in study designs, as well as in populations and outcomes studied. Moreover, the selection of studies was not fully comprehensive; instead, we chose to include a varied selection of surveys to provide as balanced a perspective as possible. The surveys originated in many different countries in Europe and North America and were conducted by different sponsors, including three different pharmaceutical companies. Nevertheless, we believe some important and consistent messages emerge that should influence better asthma management in the future.

## Conclusion

Findings of the present review of asthma surveys suggest that patients often understate their symptoms, tolerate poor symptom control, have low expectations of therapy, possess meager knowledge of correct drug usage, and display insufficient adherence to therapy. Among HCPs, there is evidence of an inadequate understanding of disease etiology and poor or unstructured communication with patients, resulting often in inaccurate assessment of disease severity. With the increasing incidence of asthma, it is important to address these many issues as a matter of priority. In particular, asthma care could be made substantially better by improving the education of patients regarding the nature of the disease (as one primarily of inflammation) and optimizing the use of existing medical systems and treatments.

## Abbreviations

HCP = healthcare provider; ICS = inhaled corticosteroids; QoL = quality of life.

## Competing interests

STH has received fees for lectures from Novartis and Merck Sharp & Dohme and is a consultant for Novartis, MRL, Almiral Prodesfarma, Rotta Pharm, Cambridge Antibody Technology, Amgen, Wyeth, UCB/Celltech, Avontec and Synairgen. DP has received honoraria for speaking at sponsored meetings from the following companies marketing respiratory products: 3 M, Altana, AstraZeneca, Boehringer Ingelheim, GlaxoSmithKline, IVAX, Merck Sharp & Dohme, Novartis, Pfizer and Schering-Plough. DP has also received honoraria for advisory panels with 3 M, Altana, AstraZeneca, Boehringer Ingelheim, GlaxoSmithKline, IVAX, MSD, Novartis, Pfizer and Schering-Plough. DP or his research team have received funding for research projects from 3 M, Altana, AstraZeneca, Boehringer Ingelheim, GlaxoSmithKline, IVAX, Merck, Sharp & Dohme, Novartis, Pfizer, Schering-Plough, Viatris. EV has received speaker's honoraria from Merck & Co.

## Figures and Tables

**Table 1:** Overall scope and dimension of the surveys

Study name	Country	Objective	Population	Design	Date
MORI/EFA [12]	France, Germany, Great Britain, Italy, Spain	Family QoL impact	Parents	Face-to-face interview. International comparison	1999–2002
TARGET [11]	Italy	Symptoms, causes, treatment	HCPs, parents	Face-to-face interviews, with physicians and patients (adults and children). Advisory board-driven	2002
AIRLife [13]	Germany	Efficacy/patient preferences	HCPs, parents	Telephone interviews with physicians and adult patients. Face-to-face interviews with asthmatic children and parents of asthmatic children	1999
National Paediatric Asthma [14]	Spain	Attitudes/QoL	Parents of young children	Face-to-face interviews with parents of children with asthma (aged 2–5 years)	2000
ASTHMA [6]	Belgium	+/- montelukast	GP/parents	GPs interviewed asthma patients before and then at least 4 weeks after treatment with montelukast	2001
ASTEQ/ASTHMA [15]	France	Symptoms while using ICS. Perception of control	Prescribers/patients/(children in subset)	Anonymous questionnaire of physicians and patients including a small pediatric substudy conducted at 20 asthma schools with 300 children (aged 2–14)	1999/2003
NOP/GPnet [16]	Great Britain	BTS guidelines impact	GPs/nurses/parents	Postal questionnaire, GPs, nurses and parents of children with asthma	2002
UK AIR [17]	Great Britain	Asthma control	GPs/nurses/children/parents	Questionnaire of patients, telephone interviews with practice nurses, face-to-face interviews of GPs	1997
Finnish AIR [18]	Finland	Asthma control	GPs/nurses/adult patients/children/parents	Postal questionnaire	2000
Danish AIR [19]	Denmark	Reality asthma control (2 year)	Patients	Postal questionnaire	2000–2002
Norwegian AIR [20]	Norway	Reality asthma control	Patients/GPs	Postal questionnaire of patients, telephone interview of GPs	2000–2001
ALMA [21]	Sweden	Reality asthma control	Patients/GPs	Telephone interviews with adult asthma patients. Questionnaire for GPs with similar questions	2000–2001
AIRE [22]	France, Great Britain, Italy, Germany, Netherlands, Spain, Sweden	How are treatment guidelines truly being applied/perceptions	Patients/children	Telephone interviews with adult asthma patients (randomly selected)	1999
ECRHS [7]	14 countries (including Canada)	Follow up on asthma development and use of services	Patients	Administered questionnaire	1990–2000
ECRHSII [23]	10 EU countries	Perception of severity, impact on society	Patients	Telephone interviews (randomly selected). Advisory-board driven	1999
Living and Breathing [24]	UK	Symptoms/control	Patients	Face-to-face interviews	2001
RESPONSE [25]	Germany, Spain, Great Britain	True symptom control, QoL, drug use	Adults/juveniles	Face-to-face interviews	2001
ACE [26]	UK	Treatment (ICS) benefit perceptions	Patients at pharmacies	Face-to-face interviews	2002
Asthma in America [27]	USA	Asthma in USA (misc.)	Adults, HCPs (physicians, nurses, pharmacists)	Telephone interviews (randomly selected)	2001
Asthma [28]	Finland	National impact snapshot	Patients	Review	1998
Psychology/Health and Medicine [29]	Sweden	Comparing assessment methodologies	Patients	Questionnaire-based survey	2003
AJN [30]	USA	Assessment/outcome tools	Children/parents	Review	2002
Illness Management Survey [31]	USA	Barriers to juvenile treatment (questionnaire support)	Juveniles	Questionnaire-based survey. Focus on compliance in highly noncompliant subset	2003
HUNAIR [32]	Hungary	Cost, morbidity, control	Children/adults	Questionnaire-based survey	1998–1999

AIR, Asthma In Real Life; BTS, British Thoracic Society; ECRHS, European Community Respiratory Health Survey; EU, European

Union; GP, general practitioner; HCP, healthcare provider; ICS, inhaled corticosteroids; QoL, quality of life.

**Table 2:** Study populations in the reviewed surveys

	Number	Percentage
Patients (adults)	45,942	69.1
Patients (children)		
Children (including juveniles)	2,820	4.2
Parents representing children	9,055	13.6
Healthcare providers		
General practitioners	4,925	7.4
Specialists	3,202	4.8
Nurses	393	0.6
Pharmacists	113	0.2
Total	66,450	100

**Table 3:** Summary of key review findings

	Subthemes	Core findings	Key supporting data
Patient perceptions	Understanding of disease	In general, patients (or caregiver) lack knowledge of their asthma and its causes	Only 22% thought asthma therapy reduced inflammation [11]
		Patients are aware of asthma symptoms, but are often willing to tolerate poor control or are unaware of the risks	92% of patients experienced limitations of activities due to asthma, and 48% had difficulty with sleeping [6]
		Despite poor control, many patients still describe themselves as 'well controlled'	>65% had symptoms during the last week, although >80% considered themselves to be 'under control' [25]
	Symptom control	Inappropriate use of available drugs may contribute to poor control	21.3% and 26.4% of patients with 'some' and 'severe' control limitations, respectively, actually used anti-inflammatory drugs [27]
		More aggressive anti-inflammatory treatment can improve control	Addition of a secondary anti-inflammatory agent (LTRA) improved sleep (87% of patients), early waking (80%), daily functionality (85%), and need for rescue medication (77%) [6]
		Patients often do not realize asthma drugs have side effects	61% of parents of children with asthma did not realize inhaled corticosteroids had side effects [16]
	Patient satisfaction	Patient satisfaction with their treatment is low	In general, these figures are understatements and inference gives higher possibilities
		Patient satisfaction (and participation) with their management is often low	28% of patients did not tell their physician in consultation about troublesome coughing, and 36% failed to mention difficulty in sleeping [15]
		Admitted compliance with treatment is often poor, expressed both by lack of as well as excessive use of prescribed treatment	45% of patients admitted using their medication excessively [19]
	Compliance	Patients cited steroid use as a major reasons for lack of compliance	One-third of patients expressed dissatisfaction with long-term steroid treatment [26]
Lifestyle issues for patients and family	Control	Lack of control was mentioned as being associated with reduced QoL in a number of surveys	General comment
	Disease severity	Correlation between QoL and disease severity was suggested	General comment
	True impact	The impact of asthma on QoL is often understated	General comment
	Lifestyle restrictions	Patients reported substantial lifestyle restrictions	Irrespective of disease severity, approximately 70% report substantial lifestyle restrictions [26]
	Families	The QoL of families of children with asthma is also clearly affected	20% of parents stated that their work attendance was affected, and 50% said their own lives were affected [14]
Child specific	Management	Generally children are better managed than adults despite some parental reservations about disease	Asthmatic children are significantly greater consumers of resources than asthmatic adults, despite having better initial asthma control [32]
	Perceptions	As in adult asthmatics, there is a marked difference between perception and reality of symptom control in children (or by their caregivers)	65% of children with asthma or their carers considered their asthma to be well controlled, although 37% had difficulty breathing, 34% had nocturnal waking, 29% had dry cough, and the ability to talk was affected in 29% at least once weekly [17]
	Therapy understanding	Parental understanding of their child's medication (and compliance) can also be poor	33% of parents of asthmatic children did not understand the role of 'controller' versus 'preventer' therapies, and only 38% of parents took their controller medication on a regular basis [12]
	Treatment needs	There seems to be a particular demand for better treatments for children	70% of parents of asthmatic children were concerned about the effects of inhaled corticosteroids [11]
Healthcare providers	Etiology	Some HCPs do not fully understand some of the recent advances in the understanding of asthma etiology	59% of physicians questioned considered allergy the main cause of asthma, with only 35% (and only 16% of pediatricians) citing the underlying inflammation. In the same survey, however, 92% of physicians understood that leukotrienes were important mediators of inflammation in asthma [11]
	Treatment needs	Some of the surveys examined physicians' inconsistent use of anti-inflammatory agents in asthma among the suboptimal numbers of patients actually being treated	92% of physicians considered anti-inflammatory drugs 'essential' in asthma care, although only 20% of patients were receiving these agents [27]
	Diagnosis	There was practical support for the need for improved diagnosis of asthma leading to improved management	The utility of decision-making tools and self-reporting questionnaires for assessing disease severity and optimizing therapy can measure and improve treatment compliance [31]
Similarities and differences between HCPs and patients	Similarities	In most relevant studies, patients and HCPs generally agreed that better treatments with fewer side effects would be desirable	General comment
	Substantial differences	HCPs and patients disagreed over symptom control	Only 1% of patients considered themselves symptom free, compared with 24% of their general practitioners [21]
		HCPs and patients disagreed over compliance levels	HCPs believed that 'all' of their patients complied with treatment, whereas only 60% of patients actually did according to HCP definition [20]
		HCPs and patients disagreed over concern towards side-effects	General comment

HCP, healthcare provider; LTRA, leukotriene receptor antagonist; QoL, quality of life.

**Table 4:** Issues in childhood asthma

Issues	Findings
Symptoms and resource use	• Children achieve a better level of symptom control than adults, and use more healthcare resources [32]
	• 72% of parents reported their children having experienced a serious asthma event [14]
Understanding of asthma and its treatment	• Only 41% of parents referred to their child's disease as 'asthma' [14]
	• 33% of parents of asthmatic children did not understand the terms 'controller' or 'preventer' therapy [12]
Impact on the life of children and family	• 21% of children had missed school within previous 3 months [12]
	• 36% of children had limitations on physical activities [12]
	• 6% of parents had missed work within previous 3 months [12]
	• 20% of parents believed their children are treated badly at school [14]
	• 50% of parents believed their lives were affected by their child's asthma [14]
Adherence	• Only 38% of parents stated that their children used controller medication regularly [12]
	• Juveniles presented particular adherence issues, showing reluctance to use inhalers in the presence of others [13]
	• In juveniles, specific decision-making tools for professionals as well as parents are helpful in identifying true severity and optimizing management [30]
	• Customized self-reported questionnaires can help identify potential noncompliance in juveniles before this became a major issue [31]
Concerns about treatments	• 70% of parents were concerned about their children using inhaled corticosteroids [11]
	• 33% of parents specified a desire for convenient nonsteroid treatments [14]
	• 66% of parents would switch their child's therapy if possible because of concerns about side effects of current drugs [14]
